# Changes in platelet maturity and reactivity following acute ST-segment elevation myocardial infarction

**DOI:** 10.1016/j.rpth.2024.102652

**Published:** 2024-12-09

**Authors:** Oliver Buchhave Pedersen, Peter H. Nissen, Leonardo Pasalic, Anne-Mette Hvas, Steen Dalby Kristensen, Erik Lerkevang Grove

**Affiliations:** 1 Thrombosis and Haemostasis Research Unit, Department of Clinical Biochemistry, Aarhus University Hospital, Aarhus, Denmark; 2 Department of Cardiology, Aarhus University Hospital, Aarhus, Denmark; 3 Department of Clinical Medicine, Faculty of Health, Aarhus University, Aarhus, Denmark; 4 Institute of Clinical Pathology and Medical Research, Westmead Hospital, New South Wales Health Pathology, Sydney, Australia; 5 Westmead Clinical School, Faculty of Medicine and Health, University of Sydney, Sydney, Australia; 6 Faculty of Health, Aarhus University, Aarhus, Denmark

**Keywords:** blood platelets, mean platelet volume, platelet activation, platelet function tests, ST elevationmyocardial infarction, SYTO-13

## Abstract

**Background:**

Reduced effect of antiplatelet therapy has been reported in patients with ST-segment elevation myocardial infarction (STEMI). This could partly be explained by an increase of highly reactive immature platelets.

**Objectives:**

To investigate changes in platelet maturity and reactivity after acute STEMI.

**Methods:**

Patients diagnosed with STEMI, admitted for primary percutaneous coronary intervention, and treated according to international guidelines, were included. Blood samples were obtained within 24 hours after admission and at 2- to 3-months follow-up. Platelet maturity and reactivity using multicolor flow cytometry with SYTO-13 to categorize platelet maturity, whole blood platelet aggregation, serum thromboxane B2 levels, and standard immature platelet markers (eg, immature platelet count and fraction, and mean platelet volume) were measured.

**Results:**

A total of 44 STEMI patients were included. The reactivity of immature platelets was consistently higher at baseline and at follow-up when compared to the entire platelet population and the mature platelet population (all *P* values < .05). The expression of CD63 (a dense granule marker) in immature platelets was consistently high compared to the entire platelet population and the mature platelet population and did not change from baseline to follow-up (*P* values > .24). Additionally, a positive significant correlation was found between standard immature platelet markers and the expression of CD63 on platelets both at baseline and follow-up (rho ranging from 0.32 to 0.62, all *P* values < .05).

**Conclusion:**

Immature platelets represent a highly reactive platelet subpopulation crucial for the overall platelet reactivity, partly due to a high expression of dense granules. Despite treatment with loading and maintenance doses of antiplatelet therapy, the reactivity of immature platelets remained high in STEMI patients.

## Essentials

• Immature platelets may affect platelet reactivity and antiplatelet therapy effectiveness.
• Platelet maturity and reactivity were assessed in ST-elevation myocardial infarction patients.
• Immature platelets are highly reactive, essential for overall platelet reactivity.
• This is partly due to a sustained high expression of dense granules after antiplatelet therapy.

## Graphical abstract

**Figure:**
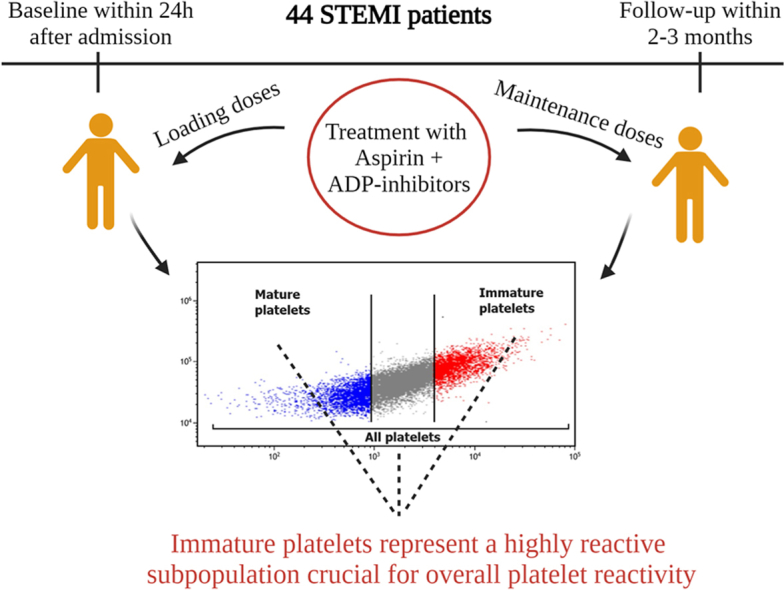

## 1 Introduction

Advancements in both mechanical and pharmacologic treatments have significantly increased the survival rate for patients with acute ST-segment elevation myocardial infarction (STEMI) [1,2]. As platelets play a pivotal pathophysiological role in arterial thrombus formation and coronary artery occlusion [3], antiplatelet therapy after acute STEMI is a crucial part of secondary prevention [4]. However, despite treatment with state-of-the-art antiplatelet therapy, many patients surviving STEMI experience recurrent cardiovascular events [5,6]. This may partly be explained by incomplete platelet inhibition observed in many STEMI patients [7,8].

Several factors may contribute to reduced effectiveness of antiplatelet drugs [9,10], including elevated platelet turnover and a relatively larger proportion of newly formed large immature platelets [11,12]. An increased platelet turnover leads to a continuous release of immature platelets that remain unaffected by irreversible antiplatelet drugs, eg, aspirin and oral P2Y12 inhibitors [12,13]. Additionally, these immature platelets may inherently constitute a more reactive platelet population when compared to mature platelets [14,15]. Immature platelets contain more granules and RNA than mature platelets, enabling protein synthesis important for platelet reactivity [15,16]. Consistent with these prothrombotic characteristics of immature platelets, prior studies have reported an increase in large and immature platelet levels during the acute phase of myocardial infarction [17,18]. Moreover, others suggested that the quantity of immature platelets may affect the risk of subsequent cardiovascular events in these patients [[19], [20], [21], [22]]. It is uncertain whether the increase in numbers of immature platelets persist after the acute event and whether this may impact the efficacy of antiplatelet therapy.

Therefore, the aim of this study was to investigate changes in platelet reactivity and the association with platelet maturity following the acute event in STEMI patients receiving antiplatelet therapy according to guidelines.

## 2 Methods

### 2.1 Study design and population

Between May 2022 and April 2023, adult patients diagnosed with STEMI [23] and admitted for coronary angiography (CAG) and primary percutaneous coronary intervention (pPCI) were included as previously reported [24]. Blood samples were obtained from the antecubital vein using a 21-gauge needle with minimal stasis at 2 distinct time points: at baseline, defined as within 24 hours after admission, and during follow-up, defined as between 2 to 3 months after the baseline sampling. Exclusion criteria were (1) the inability to give informed consent; (2) active cancer; 3) thromboembolic events or revascularization within the last 12 months; and (4) known bleeding disorder.

Additionally, for comparison, blood samples were collected from 50 healthy individuals with no history of chronic diseases and no current medical treatment, as previously described [25].

Approval for the study was granted by the Central Denmark Region Committees in Biomedical Research Ethics (Reference number: 1–10–72-30-21) and the Danish Data Protection Agency (Journal number: 1–16–02-349-21). All participants provided informed oral and written consent, and the study complied with the principles outlined in the Helsinki-II Declaration.

### 2.2 Platelet reactivity markers determined with manual flow cytometry

Blood samples were collected in sodium citrate 3.2% tubes (Terumo Europe) and handled as previously described [26]. Platelet reactivity was evaluated employing a combination of fluorescence-labeled antibodies targeting the following platelet surface markers: CD63-PECy7 (mouse antibody, clone H5C6, Nordic BioSite), P-selectin-PE (CD62P) (mouse antibody, clone AK4, Nordic BioSite) and bound-fibrinogen-V420 (antifibrinogen) (polyclonal chicken, Diapensia HB). Single platelets were identified with CD42b-AF700 (a platelet-specific marker, mouse antibody, clone HIP1, Nordic BioSite) and using CD45-BV650 (a leukocyte-specific marker, mouse antibody, clone HI30, Nordic BioSite) to exclude leukocytes. Additionally, we also employed the SYTO-13 dye (ThermoFisher Scientific) to differentiate between immature and mature platelets [25,26]. Platelets were classified and analyzed across 3 groups: (1) all platelets; (2) the 20% of platelets with the highest SYTO-13 content, indicative of elevated platelet RNA levels (SYTO-high) corresponding to the most immature platelets; and (3) the 20% of platelets with the lowest SYTO-13 content and platelet RNA levels (SYTO-low) corresponding to the most mature platelets. Platelets were activated using thrombin-receptor-activating-peptide-6 (TRAP) (28.6 μM, JPT), adenosine diphosphate (ADP) (10.8 μM, Sigma-Aldrich) and collagen-related peptide (CRP) (0.05 μg/mL, University of Cambridge). Flow cytometry was conducted using a four-laser CytoFLEX S (B75408) flow cytometer (Beckman Coulter). Positive gates were set to include 1% to 2% events for antifibrinogen and CD63, 0.1% to 0.2% events for P-selectin, and 0.02% to 0.04% events for SYTO-13 on the negative control consisting of the same antibodies except for P-selectin-PE, which was replaced with a matching isotype control, and 5 mL of 6mM EDTA in dilution buffer was used instead of agonist [26,27]. A minimum of 15,000 single platelets were acquired for each sample. An acceptable pre-activation threshold was set at 15%, assessed by measuring the percentage of P-selectin–positive platelets in a sample without agonist addition, to determine preactivation during antibody preparation [26]. Additional settings followed the MIFlowCyt guideline [28] as described previously [26,27]. Daily quality control using fluorescent QC beads (CytoFLEX Daily QC Fluorophores, Beckman Coulter) was performed as per manufacturer’s instructions. Platelet reactivity was assessed by median fluorescence intensity of the reactivity markers (MFI) and the percentage of platelets positive for the reactivity markers (%Gated).

### 2.3 Other laboratory measurements

Platelet count, immature platelet count (IPC), immature platelet fraction (IPF), mean platelet volume (MPV), platelet distribution width (PDW), platelet large-cell-ratio (P-LCR), white blood cell count, and hemoglobin levels were quantified in tubes containing EDTA (Becton Dickinson Bioscience) using a standard diagnostic hematology analyzer (Sysmex XN-9000). Plasma creatinine, estimated glomerular filtration rate, and C-reactive protein were analyzed in lithium-heparin tubes (Becton Dickinson Bioscience) with the fully automated Atellica CH system (Siemens). Plasma fibrinogen was analyzed in sodium citrate 3.2% tubes (Terumo Europe) using the CS-5100 automated coagulation system (Sysmex).

Whole blood platelet aggregation was assessed with whole blood impedance aggregometry employing the Multiplate Analyzer (Roche Diagnostics A/S). Platelets were activated with the following agonists: ADP (ADPtest 6.5, μM), TRAP (TRAPtest, 32 μM), and arachidonic acid (AA) (ASPItest, 0.5 mM).

Serum thromboxane B_2_ (TXB_2_) levels were measured using ELISA according to the manufacturer’s instructions (Cayman Chemical).

### 2.4 Statistical analysis

Data distribution was evaluated using Q-Q plots and histograms. If normally distributed, continuous data are presented as mean and SD, if not as median and IQR. Categorical data are presented by percentages. Differences were tested with paired Wilcoxon signed rank sum test. Correlation analysis between platelet maturity markers and platelet reactivity was performed using Spearman’s rho for data not following normal distribution and illustrated using heatmaps. A formal sample size calculation was not performed due to the explorative nature of the study. All tests of significance were two-tailed with a probability value of *P* <.05. All analyses were performed in Rstudio (Integrated Development for R, PBC) and GraphPad Prism 9 (GraphPad Software Inc).

## 3 Results

### 3.1 Study population

Among the 61 acute STEMI patients enrolled with baseline measurements [24], follow-up samples were obtained in 44 patients. Hence, the present study included 44 acute STEMI patients with a completed study protocol. No differences were found in demographics, cardiovascular risk factors, initiated treatments, or baseline biochemistry and hematological values between patients with (*n* = 44) or without follow-up (*n* = 17) (data not shown).

Of the 44 included STEMI patients, 20% were females, the median age was 62 years and 7% had prior ischemic heart disease. The median time from baseline to follow-up was 67 days (Table 1). C-reactive protein and leukocyte values were significantly lower at follow-up than at baseline (Table 1). Furthermore, IPF was statistically significantly lower at follow-up than in the acute phase of STEMI (3.8 [2.5, 6.0] vs = 4.4 [2.9, 6.5]; *P* = .03), whereas no difference in other immature platelet and platelet turnover markers was observed (Table 1). No patients experienced a recurrent cardiovascular event during the study period.

**Table 1:** Characteristics of the study population consisting of 44 patients with acute ST-segment elevation myocardial infarction (STEMI).

Demographics and risk factors	Values		
Female sex, *n* (%)	9 (20)		
Age (y)	62 (51;69)		
Caucasian	44 (100)		
Body mass index (kg/m^2^)	28 (26;31)		
Diabetes mellitus, *n* (%)	10 (23)		
Smoking, *n* (%)	19 (43)		
Hypertension, *n* (%)	23 (52)		
Hypercholesterolemia, *n* (%)	17 (39)		
Prior ischemic heart disease, *n* (%)	3 (7)		
Time from admission to baseline (h)	13 (9;16)		
Time from baseline to follow-up (d)	67 (59;73)		

**Medications**	**Values**		
Statins, *n* (%)	33 (75)		
ACE-inhibitor, n (%)	7 (16)		
AT II-antagonist, *n* (%)	14 (32)		
Diuretics, *n* (%)	6 (14)		
Calcium-antagonist, *n* (%)	12 (27)		
DOAC, *n* (%)	1 (2)		

**Biochemistry and hematology**	**Baseline**	**Follow-up**	***P* value**^a^
Creatinine (μmol/L)	72 (62;78)	73 (64;84)	.2
eGFR (mL/min)	90 (88;90)	90 (84;90)	.1
C-reactive protein (mg/L)	5 (4;20)	4 (4;4)	< .0001
Leukocytes (×10^9^/L)	11 (9;13)	7 (6;8)	< .0001
Hemoglobin (mmol/L)	8.9 (8.4;9.4)	8.9 (8.5;9.3)	.4
Platelet count (×10^9^/L)	254 (231;290)	274 (240;296)	.2
Fibrinogen (μmol/L)	11.5 (10.4;12.5)	11.3 (9.2;12.6)	.2
Platelet count (×10^9^/L)	254 (231;290)	274 (240;296)	.2
Immature platelet count (×10^9^/L)	10.2 (8.1;17)	10.2 (7.1;15.4)	.2
Immature platelet fraction (%)	4.4 (2.9;6.5)	3.8 (2.5;6.0)	.03
Mean platelet volume (fL)	9.8 (9.1;10.6)	9.7 (9.1;10.4)	.1
Platelet distribution width (fL)	11.2 (10;13)	10.9 (9.6;12.4)	.07
Platelet large-cell-ratio, *n* (%)	0.2 (0.2;0.3)	0.2 (0.2;0.3)	.3

All values are median (interquartile range) unless stated otherwise.

ACE, angiotensin-converting enzyme; AT II, angiotensin II; DOAC, direct oral anticoagulant; eGFR, estimated glomerular filtration rate.

a Using the paired Wilcoxon signed rank test.

A total of 29 patients (66%) had single-vessel disease, 9 patients (20%) had two-vessel disease, and 6 patients (14%) had three-vessel disease, as shown in Table 2. Following pPCI procedures, 29 patients (66%) achieved full revascularization at the index percutaneous coronary intervention (PCI), while 7 patients (16%) and 4 patients (9%) underwent staged PCI or coronary artery bypass graft surgery during hospitalization (Table 2).

**Table 2:** Coronary angiogram findings and procedures in 44 patients with acute ST-segment elevation myocardial infarction (STEMI).

Variables, all indicated as *n* (%)	Values
Number of vessel affected	
1-vessel disease	29 (66)
2-vessel disease	9 (20)
3-vessel disease	6 (14)
Vessel affected	
LM	3 (7)
LAD	24 (55)
LCX	11 (25)
RCA	28 (64)
Fully revascularized at index PCI	29 (66)
Fully revascularized after staged PCI during hospitalization	7 (16)
Fully revascularized after index PCI and CABG during hospitalization	4 (9)
Primary PCI and incomplete revascularization (OMT)	4 (9)

LM, left main; LAD, left anterior descending artery; LCX, left circumflex artery; RCA, right coronary artery; PCI, primary percutaneous intervention; CABG, coronary artery bypass graft surgery; OMT, optimal medical treatment.

At the time of pPCI, patients were treated with antiplatelet and anticoagulant therapy according to international guidelines, Table 3 [4]. Patients were treated with loading doses of antiplatelet treatment during or immediately following intervention consisting of 60 mg prasugrel (82%), 600 mg clopidogrel (9%) or 180 mg ticagrelor (9%), Table 3. After loading doses, patients were treated with the recommended maintenance doses (Table 3).

**Table 3:** Antithrombotic treatment in 44 patients with ST-segment elevation myocardial infarction.

Baseline sampling	*n* (%)
Preinterventional treatment	
Aspirin (300-mg loading dose)	43 (98)
Unfractionated heparin (weight-based)	41 (98)
Peri-interventional treatment	
Cangrelor	12 (27)
Postinterventional antiplatelet treatment (loading dose)	
Prasugrel (60 mg)	36 (82)
Clopidogrel (600 mg)	4 (9)
Ticagrelor (180 mg)	4 (9)

**Follow-up sampling (maintenance dose)**	
Aspirin (75 mg × 1 daily)	43 (98)
Prasugrel (10 mg × 1 daily)	32 (73)
Prasugrel (5 mg × 1 daily)	4 (9)
Clopidogrel (75 mg × 1 daily)	4 (9)
Ticagrelor (90 mg × 2 daily)	4 (9)

### 3.2 Platelet maturity and platelet reactivity

**Figure 1:**
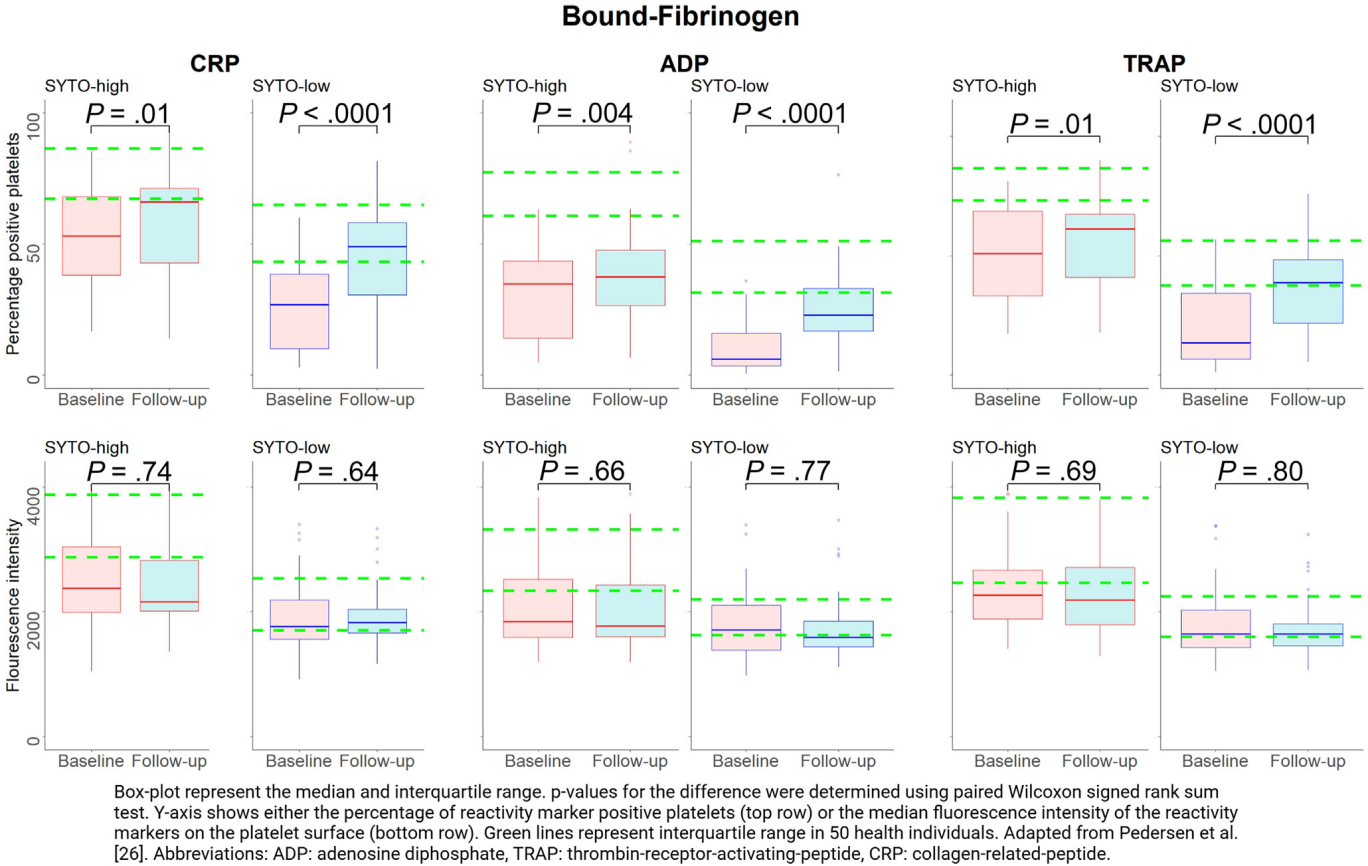
Changes in bound-fibrinogen expression on activated platelets from baseline to follow-up in patients with ST-segment elevation myocardial infarction (STEMI) in the 20% SYTO-high platelets (corresponding to immature platelets), and the 20% SYTO-low platelets (corresponding to mature platelets). Adapted from Pedersen et al [26]. ADP, adenosine diphosphate; CRP, collagen-related peptide; TRAP, thrombin-receptor-activating-peptide-6.

**Figure 2:**
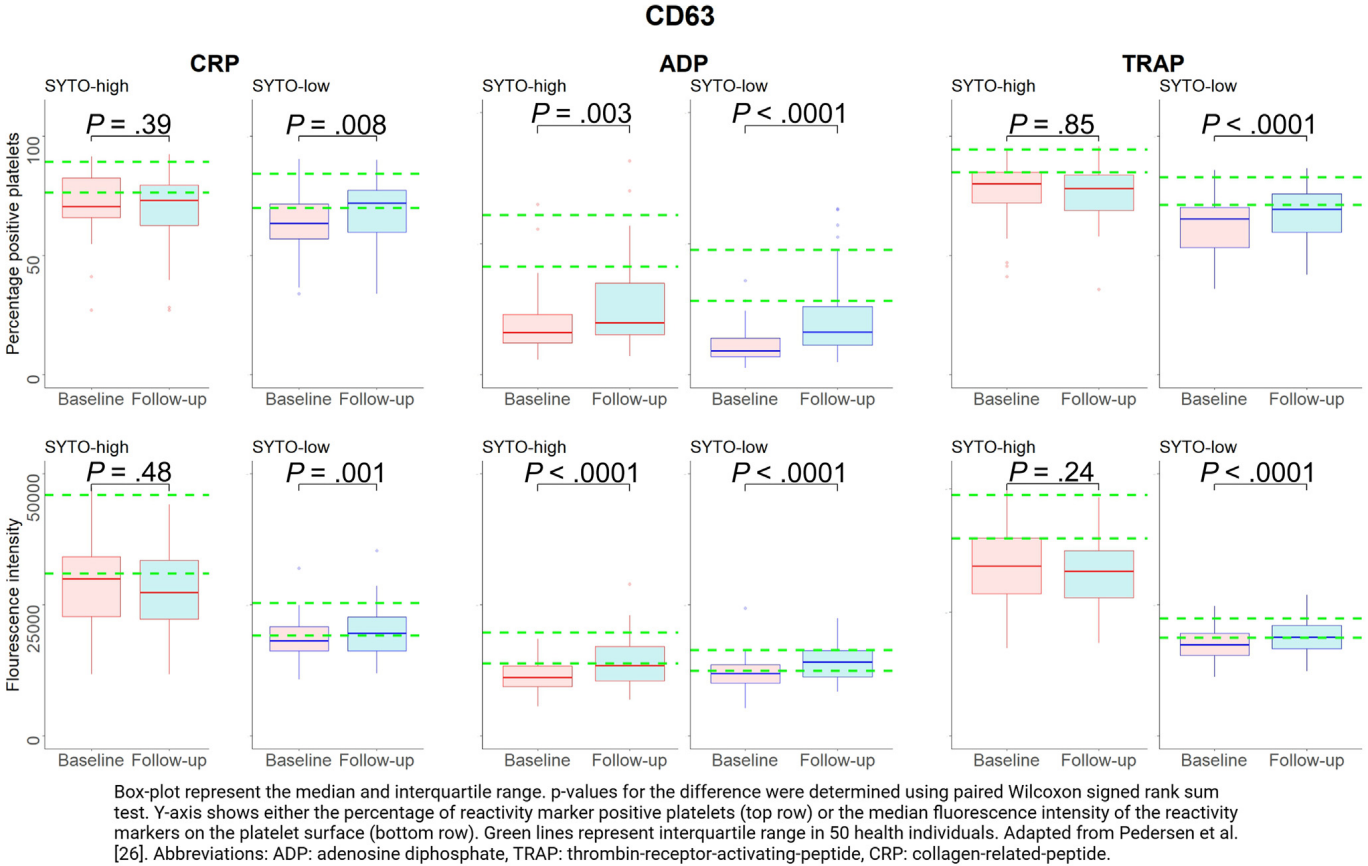
Changes in CD63 expression on activated platelets from baseline to follow-up in patients with ST-segment elevation myocardial infarction (STEMI) in the 20% SYTO-high platelets (corresponding to immature platelets), and the 20% SYTO-low platelets (corresponding to mature platelets). Adapted from Pedersen et al [26]. ADP, adenosine diphosphate; CRP, collagen-related peptide; TRAP, thrombin-receptor-activating-peptide-6.

**Figure 3:**
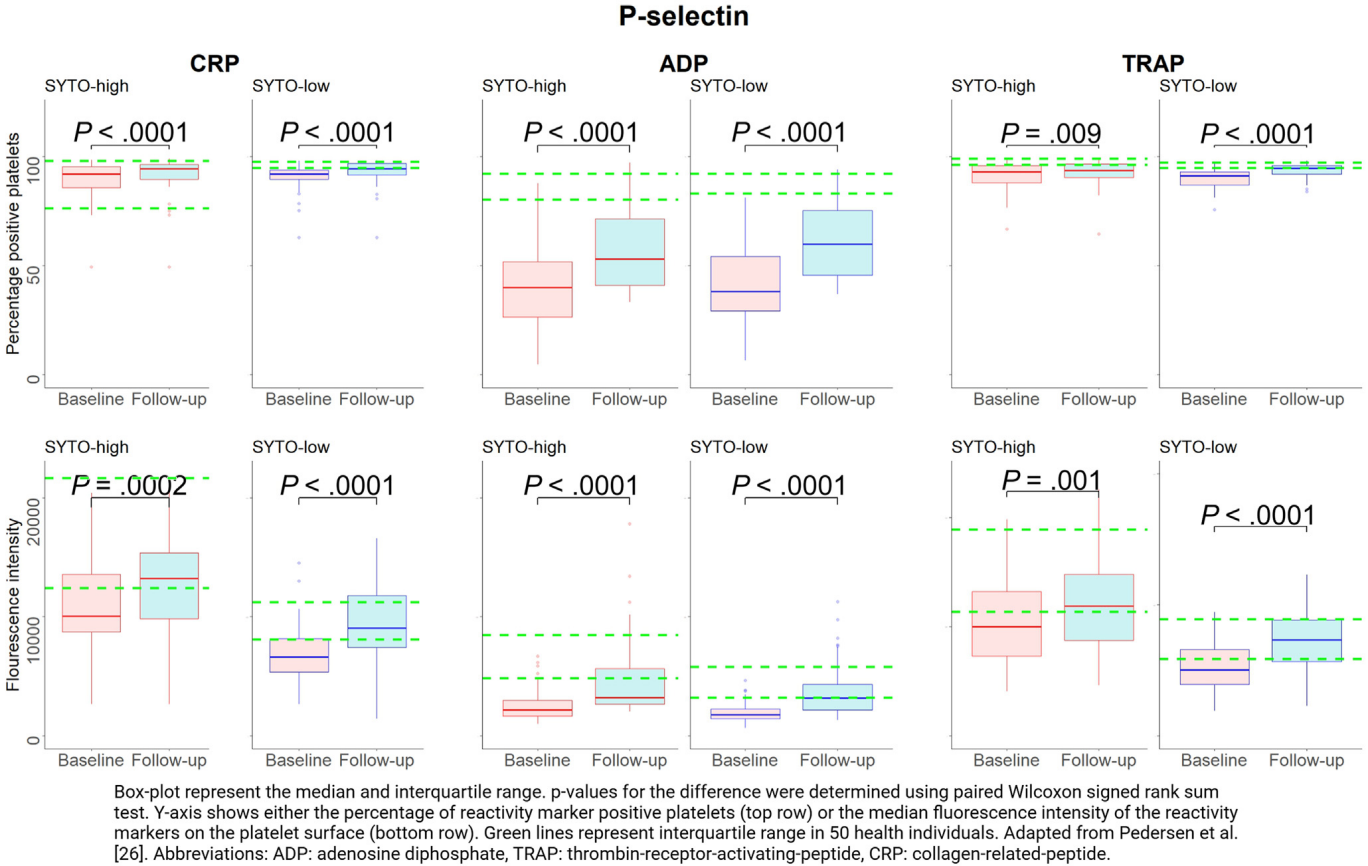
Changes in P-selectin expression on activated platelets from baseline to follow-up in patients with ST-segment elevation myocardial infarction (STEMI) in the 20% SYTO-high platelets (corresponding to immature platelets), and the 20% SYTO-low platelets (corresponding to mature platelets). Adapted from Pedersen et al [26]. ADP, adenosine diphosphate; CRP, collagen-related peptide; TRAP, thrombin-receptor-activating-peptide-6.

Flow cytometric investigation of platelet reactivity was analyzed in 3 platelet groups: (1) all platelets; (2) immature platelets (SYTO-high platelets); and (3) mature platelets (SYTO-low platelets). Overall, we found low preactivation at both baseline (5.6% [3.1, 8.8]) and follow-up 7.8% [5.1, 11.7]). Immature platelets had consistently higher platelet reactivity at both baseline and follow-up than all platelets and mature platelets (Figure 1, Figure 2, Figure 3 and Supplementary Figure S1–S3).

In immature platelets, the percentage of platelets positive for bound-fibrinogen was significantly higher at follow-up than at baseline regardless of the agonist used (all *P* values < .01; Figure 1). No differences in fluorescence intensity of bound-fibrinogen expression between baseline and follow-up were observed (all *P* values > .66; Figure 1). The percentage of positive platelets and the fluorescence intensity of CD63 did not change from baseline to follow-up when CRP or TRAP were used as agonists (all *P* values > .24), whereas these values were significantly higher at follow-up than at baseline when using ADP as agonist (*P* values < .003; Figure 2). Both the percentage of positive platelets and the fluorescence intensity of P-selectin were significantly higher at follow-up than at baseline for all agonists (all *P* vales < .001; Figure 3).

In mature platelets, the percentage of platelets positive for bound-fibrinogen was significantly higher at follow-up than at baseline regardless of the agonist used (all *P* values < .0001; Figure 1). No differences in the fluorescence intensity of bound-fibrinogen expression between baseline and follow-up were observed (all *P* values > .64; Figure 1). The percentage of positive platelets and the fluorescence intensity of CD63 were significantly higher at follow-up than at baseline for all used agonists (all *P* values < .008; Figure 2). Both the percentage of positive platelets and the fluorescence intensity of P-selectin were significantly higher at follow-up than at baseline regardless of used agonist (all *P* values < .0001; Figure 3).

In all platelets, the percentage of platelets positive for bound-fibrinogen was significantly higher at follow-up than at baseline regardless of the agonist used (all *P* values < .0001; Supplementary Figure S1). No differences in the fluorescence intensity of bound-fibrinogen expression between baseline and follow-up were observed (*P* values ranging from .59 to .74; Supplementary Figure S1). Both the percentage of positive platelets and fluorescence intensity of CD63 were significantly higher at follow-up than at baseline for all used agonists (all *P* values < .02), except for the percentage of positive platelets using CRP as agonist (*P* = .25; Supplementary Figure S2). Similarly, both the percentage of positive platelets and the fluorescence intensity of P-selectin were significantly higher at follow-up than at baseline regardless of the type of agonist (all *P* values < .0001; Supplementary Figure S3).

**Figure 4:**
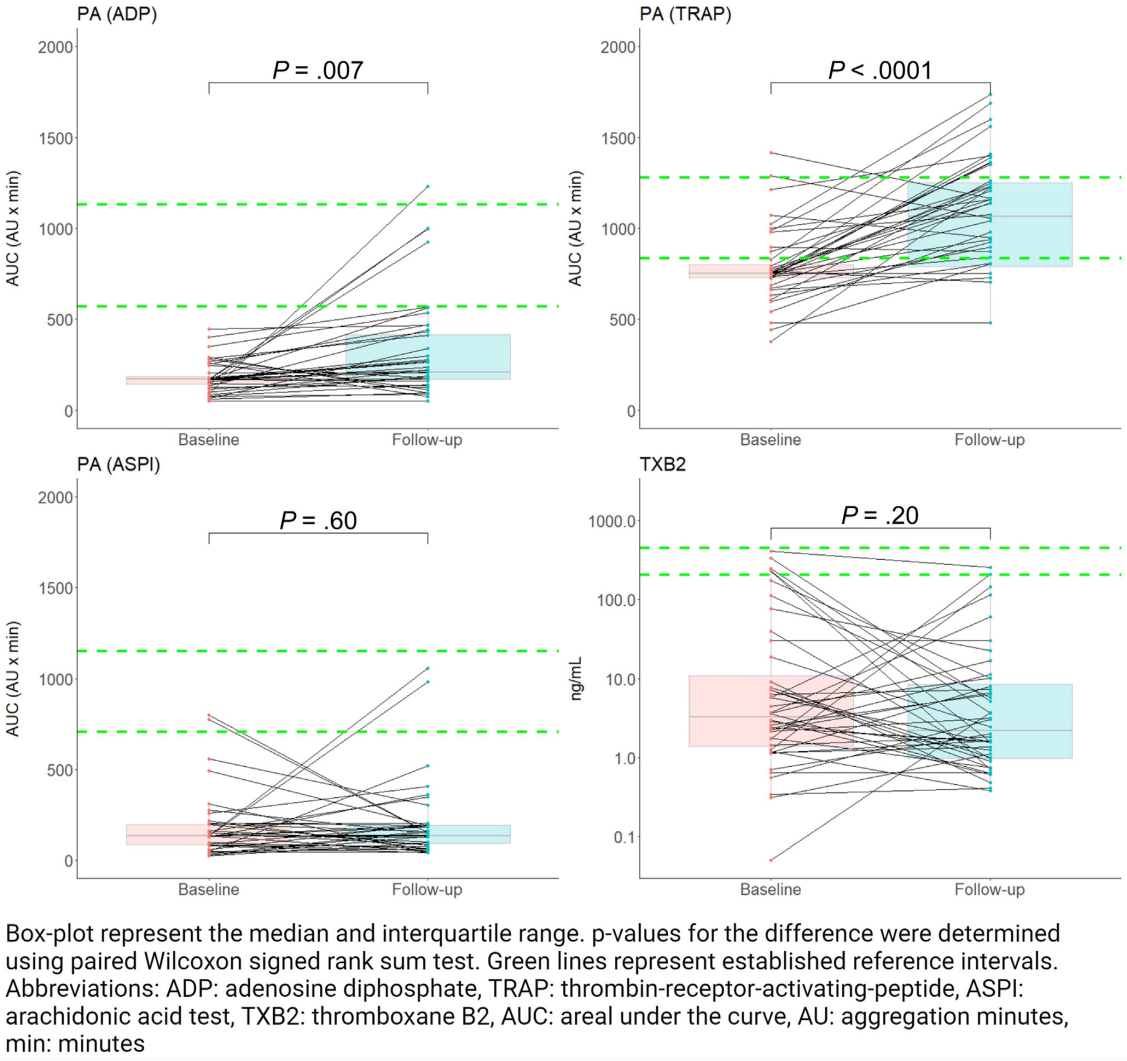
Changes in platelet aggregation and serum thromboxane B2 (TXB_2_) from baseline to follow-up in 44 patients with ST-segment elevation myocardial infarction (STEMI). ADP, adenosine diphosphate; ASPI, arachidonic acid test; AU, aggregation minutes; AUC, area under the curve; CRP, collagen-related peptide; TRAP, thrombin-receptor-activating-peptide-6, TXB2, thromboxane B2;

At follow-up, platelet aggregation using ADP or TRAP as agonists was significantly higher than at baseline (*P* = .007 and *P* < .0001, respectively; Figure 4). Platelet aggregation using AA as agonist and serum TXB_2_ levels were unchanged (*P* = .60 and *P* = .20, respectively; Figure 4).

Overall, we found a statistically significant positive correlation between standard immature platelet markers (IPC, IPF, MPV, P-LCR, and PDW) and the fluorescence intensity (MFI) of CD63 expression at baseline (rho ranging from 0.31 to 0.62, all *P* values < .04; Supplementary Figure S4). At follow-up, we also observed a statistically significant positive correlation between standard immature platelet markers and the fluorescence intensity of CD63 regardless of the used agonist, Supplementary Figure S5 (rho ranging from 0.30 to 0.58, all *P* values < .05).

### 3.3 Effect of antiplatelet therapy

Using a serum TXB_2_ level of 10 ng/mL as a cutoff below which the pharmacologic effect of aspirin corresponds to 95% COX-1 inhibition [29], and 11 STEMI patients had TXB_2_ levels above this cutoff at the time of acute STEMI. In these 11 STEMI patients, a reduction in TXB_2_ levels was observed in all patients at follow-up, with TXB_2_ levels remaining above the cutoff in only 4 STEMI patients (Supplementary Figure S6). No differences in other examined variables were observed including standard immature platelet markers between the 4 STEMI patients with TXB_2_ level above the cutoff and STEMI patients below the cutoff (all *P* values > .05). In addition, 11 STEMI patients had TXB_2_ levels above 10 ng/mL at follow-up. There were no differences in investigated variables between STEMI patients with high TXB_2_ levels and STEMI patients with suppressed TXB_2_ levels (all *P* values > .05). Only platelet aggregation with AA as agonist was significantly higher in patients with high TXB_2_ levels than in patients with suppressed TXB_2_ levels (data not shown).

A maintenance dose of 10 mg of prasugrel was used in 36 patients and a maintenance dose of 5 mg prasugrel, ticagrelor, and clopidogrel was only utilized in 4 patients each. Using a cutoff to define reduced effect of ADP-inhibitors with impedance platelet aggregation of over 46 aggregation units [30] with ADP as agonist, we identified 9 STEMI patients with values above this threshold at follow-up. Of these 9 patients with high platelet aggregation with ADP as agonist, 4 were treated with clopidogrel, 1 was treated with ticagrelor, 1 was treated with 5 mg prasugrel, and 3 were treated with 10 mg prasugrel. Patients with high platelet aggregation with ADP as agonist at follow-up had higher values of standard immature platelet markers than patients with low platelet aggregation at follow-up (Supplementary Table S1). Additionally, patients with high platelet aggregation with ADP as agonist at follow-up had higher platelet reactivity using multicolor flow cytometry, platelet aggregation with AA, and TRAP as agonists and serum thromboxane B2 levels (Supplementary Table S1).

A total of 12 patients (27%) received cangrelor during the PCI procedure. We observed no significant differences in platelet reactivity, platelet aggregation, TXB_2_ levels, or traditional cardiovascular risk factors between STEMI patients treated with cangrelor and those not treated with cangrelor (all *P* values > .05).

## 4 Discussion

In a recent study [25], we used for the first time a multicolor flow cytometry technique with SYTO-13 dye to classify platelets as immature or mature in STEMI patients and in healthy individuals, and subsequently investigated the reactivity of these platelet populations. The present study is the first to investigate changes in platelet reactivity within platelet subpopulations using this multicolor flow cytometric method in patients after acute STEMI treated according to international guidelines.

Our main finding was that immature platelets constitute a highly reactive platelet subpopulation in STEMI patients, maintaining their high reactivity even after loading dose and also after maintenance doses of antiplatelet therapy. This high platelet reactivity was observed in all measured expression levels of the platelet reactivity markers (CD63, P-selectin, and bound-fibrinogen) regardless of the agonist applied (CRP, ADP, and TRAP). This adds to findings from previous studies reporting increased reactivity of immature platelets in healthy individuals [31,32] and in patients with high platelet turnover [15,32]. Additionally, studies have demonstrated that the amount of immature platelets [18,33] and platelet reactivity are increased in acute STEMI [34], suggesting a significant role of immature platelets for thrombus formation in acute coronary syndromes [12,18,33].

Despite being a highly reactive platelet subpopulation, the expression of reactivity markers on immature platelets was generally lower than in healthy individuals [25], indicating that antiplatelet therapy has an inhibitory effect on the reactivity of all platelets including the highly reactive immature platelets. Notably, we observed that the overall inhibitory effect of antiplatelet therapy on the expression levels of platelet reactivity markers was more pronounced after loading doses than during maintenance doses of antiplatelet therapy. This finding may suggest that a higher maintenance dose of antiplatelet therapy or a shortening of the dosing interval is needed to counteract the continuous release of immature platelets, which remain unaffected by irreversible antiplatelet drugs in conditions with increased platelet turnover as STEMI [12,13]. However, during the acute event, circulating platelets may have already been activated and released their granules, which could explain the significantly lower expression levels of reactivity markers observed at baseline. Finally, other factors may partly contribute to this finding, including compliance issues in patients on maintenance antiplatelet therapy [10]. However, we found an overall low serum TXB_2_ value, indicating that patients were compliant with their medication, at least with aspirin [35]. The finding of a more pronounced effect of antiplatelet therapy after loading doses than after maintenance doses was shown for measurements in all platelets and also in the mature platelet subpopulation. Additionally, this was also observed in immature platelets regarding the expression of bound-fibrinogen and P-selectin, but not in the expression of CD63 (except when using ADP as agonist). The observed differences in flow cytometry results were consistent when reported as both and %Gated, except for bound-fibrinogen, where %Gated differed between baseline and follow-up, while no difference was observed in MFI. This discrepancy could be attributed to several factors, both technical and biological. However, as quality control measures were performed before each analysis to maintain consistent settings throughout the study, technical variations likely contributed minimally to these findings. From a biological perspective, this observation may suggest that certain platelet subpopulations increased either the proportion of platelets expressing specific surface markers or the quantity of those markers per platelet. Therefore, this finding underscores the importance of reporting both MFI and %Gated parameters in flow cytometry analyses.

We found a strong correlation between standard immature platelet markers and the expression of CD63 at both baseline and follow-up in STEMI patients. The persistent high expression of CD63 on immature platelets, even during treatment with dual antiplatelet therapy, suggests a permanent substantial presence of dense granules [36]. This finding aligns with ultrastructural observations of immature platelets showing increased quantity of dense granules [37]. Dense granules within platelets are rich in ADP, which plays a crucial role in enhancing platelet aggregation by binding to P2Y1 and P2Y12 receptors [36]. Elevated ADP levels, along with increased intracellular calcium from platelet activation, promote the secretion and production of platelet agonists like thromboxane A2 (TXA2) [38,39]. TXA2 induces vasoconstriction and stimulates platelet activation, enhancing aggregation and facilitating the formation of a stable platelet plug [40]. As immature platelets contain a high level of dense granules as we also found in the present study, this may partly explain the hypothesis that antiplatelet therapy is less effective on immature platelets [[41], [42], [43]]. An increased number of immature platelets signifies elevated platelet production, resulting in an ongoing release into the circulation of new platelets that remain unaffected by antiplatelet therapy [[44], [45], [46]]. This persistent production of new and highly reactive platelets may undermine the effectiveness of antiplatelet therapy, potentially resulting in significant clinical consequences [[44], [45], [46]]. In the present study, we found no difference in standard immature platelet markers between baseline and follow-up measurements. This is contrary to previous studies demonstrating increased platelet production and amount of immature platelets in acute STEMI [18,47].

At baseline, we observed 11 acute STEMI patients with elevated serum-TXB_2_ levels, indicating a possible reduced effect of aspirin in these patients [29]. However, there were no difference in any other measured parameters of platelet reactivity or traditional cardiovascular risk factors between acute STEMI patients with elevated TXB_2_ levels and patients with low TXB_2_ levels. At follow-up, TXB_2_ levels remained elevated in only 4 patients, with all patients showing lower TXB_2_ levels at follow-up than at baseline. Aspirin only inhibits a single pathway of platelet activation, and the importance of this pathway varies considerably among individuals [48]. We identified 9 patients with platelet aggregation using ADP as agonist above suggested cutoff to define reduce effect of ADP-inhibitors [30] at follow-up. Interestingly, we observed that patients exceeding this cutoff exhibited higher levels of standard immature platelet markers than those below it. This suggests that immature platelets might compromise the effectiveness of antiplatelet therapy [42,44]. Other factors, in particular compliance, may also be important [9,10].

Large clinical trials investigating the benefits of platelet-reactivity–guided and tailored antiplatelet therapy have not demonstrated significant improvement in clinical outcomes [[49], [50], [51], [52]]. These studies predominately used the same platelet function test (VerifyNow) and primarily used ADP as agonist [[49], [50], [51], [52]]. A more comprehensive approach with different assays to evaluate the effect of antiplatelet therapy, including the use of multiple agonists and repeated assessments of platelet reactivity, may be necessary to improve personalized treatment efficacy and patient outcomes in STEMI patients. Additionally, future studies should investigate whether certain patient groups, such as those with increased platelet turnover, may benefit from treatment with reversible ADP inhibitors (eg, ticagrelor) to counteract the continuous release of immature platelets into the bloodstream.

The main strength of the present study is the well-characterized study population treated according to international guidelines, and the wide-ranging use of methods to investigate platelet maturity and reactivity, including a multicolor flow cytometric method with SYTO-13 dye. However, certain limitations should be considered. Some STEMI patients received opiates during the acute phase, which may delay the absorption of oral ADP inhibitors [53]. Unfortunately, we did not register which patients received opiates, but it may have influenced the observed platelet reactivity after loading doses of ADP inhibitors. As most patients included were treated with prasugrel, we could not explore potential differences in platelet reactivity and immature platelets depending on the type of ADP inhibitor. The findings presented in this study may not be directly extrapolated to patients with different types of myocardial infarction, as our analysis focused on STEMI patients. We collected 15,000 platelets per sample for our flow cytometry analysis and categorized immature and mature platelet subpopulations based on the top 20% and bottom 20% of SYTO-13 content, respectively. With only 3000 platelets per subpopulation, small changes in positivity may have influenced results, especially for measurements with relatively minor differences between baseline and follow-up. Finally, due to the exploratory nature of this study, we did not perform a formal sample size calculation.

In conclusion, immature platelets represent a highly reactive subpopulation crucial for overall platelet reactivity. This is partly due to their high content of dense granules, which are important for platelet reactivity and remain elevated in immature platelets despite treatment with loading and maintenance doses of antiplatelet therapy. As many STEMI patients experience reduced effect of antiplatelet therapy, more studies are needed to investigate whether antiplatelet therapy should be tailored in patients with an increased number of highly reactive immature platelets.
