# Supplementary material for: Changes in platelet maturity and reactivity following acute ST-segment elevation myocardial infarction
Source: Res Pract Thromb Haemost. 2024 Dec 9;9(1):102652. doi: 10.1016/j.rpth.2024.102652 (PMC11751529; doi:10.1016/j.rpth.2024.102652)
Supplement: Supplementary Material [file mmc1.pdf]

Supplementary Figure S1. Changes in bound-fibrinogen expression on activated platelets from baseline to follow-up in patients with ST-segment elevation myocardial infarction (STEMI) in categories of all platelets, 20% SYTO-high platelets (corresponding to immature platelets), and 20% SYTO-low platelets (corresponding to mature platelets).

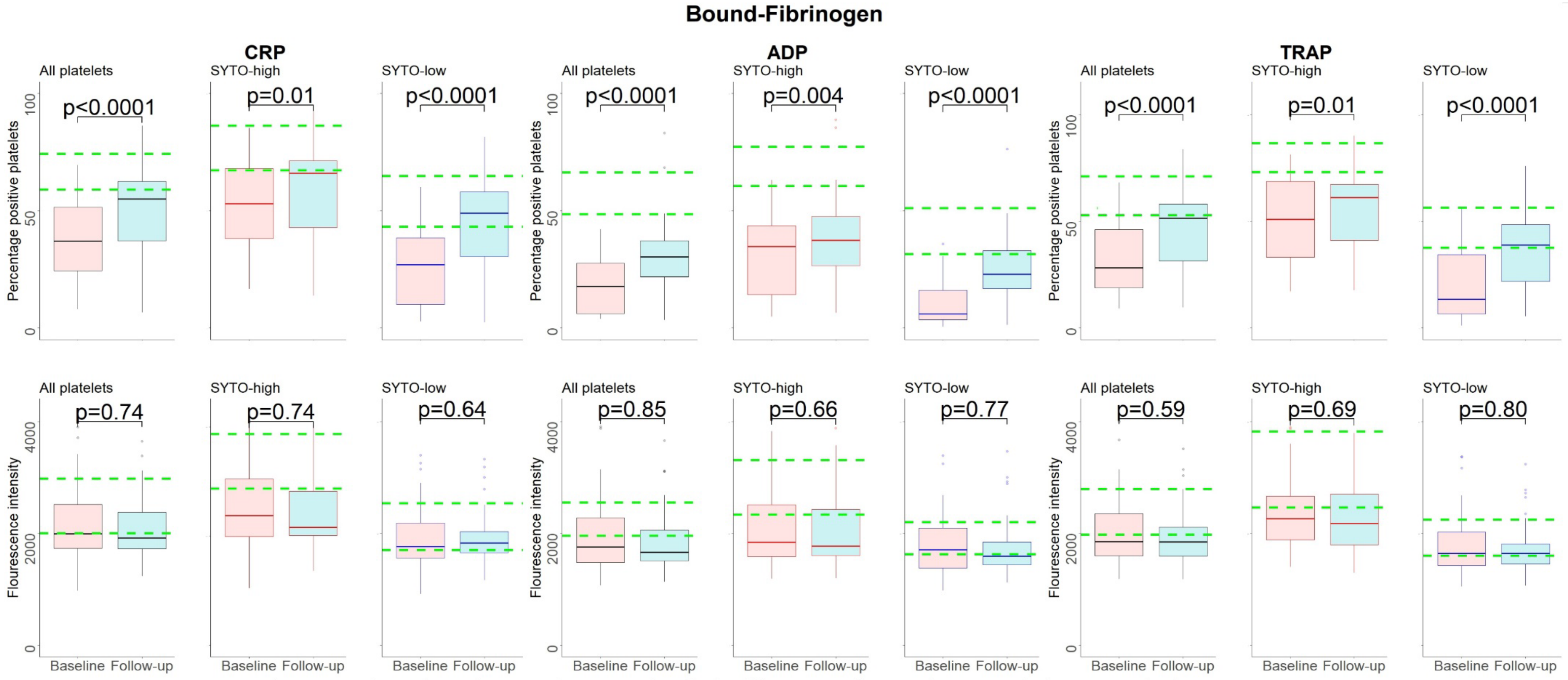

Box-plot represent the median and interquartile range. p-values for the difference were determined using paired Wilcoxon signed rank sum test. Y-axis shows either the percentage of reactivity marker positive platelets (top row) or the median fluorescence intensity of the reactivity markers on the platelet surface (bottom row). Green lines represent interquartile range in 50 health individuals. Adapted from Pedersen et al. [26]. Abbreviations: ADP: adenosine diphosphate, TRAP: thrombin-receptor-activating-peptide, CRP: collagen-related-peptide.

Supplementary Figure S2. Changes in CD63 expression on activated platelets from baseline to follow-up in patients with ST-segment elevation myocardial infarction (STEMI) in categories of all platelets, 20% SYTO-high platelets (corresponding to immature platelets), and 20% SYTO-low platelets (corresponding to mature platelets).

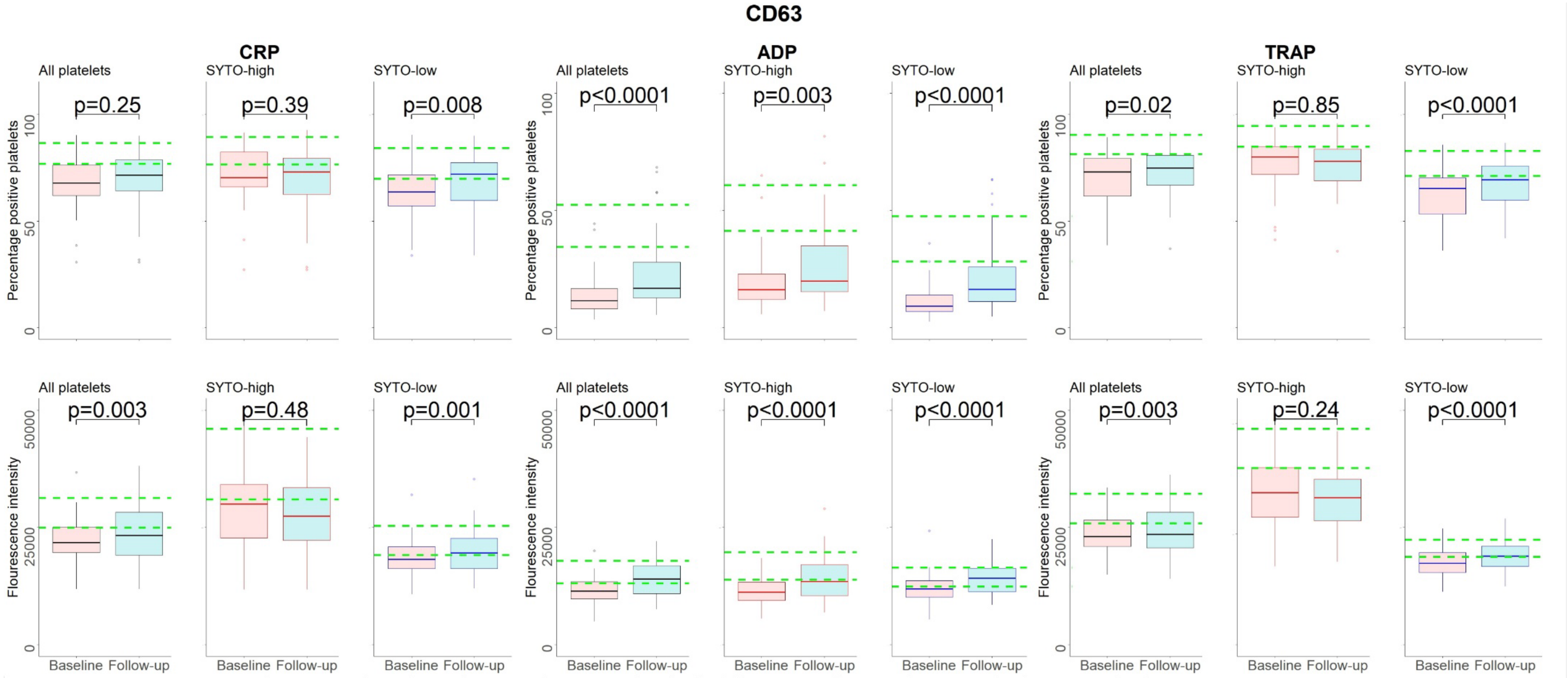

Box-plot represent the median and interquartile range. p-values for the difference were determined using paired Wilcoxon signed rank sum test. Y-axis shows either the percentage of reactivity marker positive platelets (top row) or the median fluorescence intensity of the reactivity markers on the platelet surface (bottom row). Green lines represent interquartile range in 50 health individuals. Adapted from Pedersen et al. [26]. Abbreviations: ADP: adenosine diphosphate, TRAP: thrombin-receptor-activating-peptide, CRP: collagen-related-peptide.

Supplementary Figure S3. Changes in P-selectin expression on activated platelets from baseline to follow-up in patients with ST-segment elevation myocardial infarction (STEMI) in categories of all platelets, 20% SYTO-high platelets (corresponding to immature platelets), and 20% SYTO-low platelets (corresponding to mature platelets).

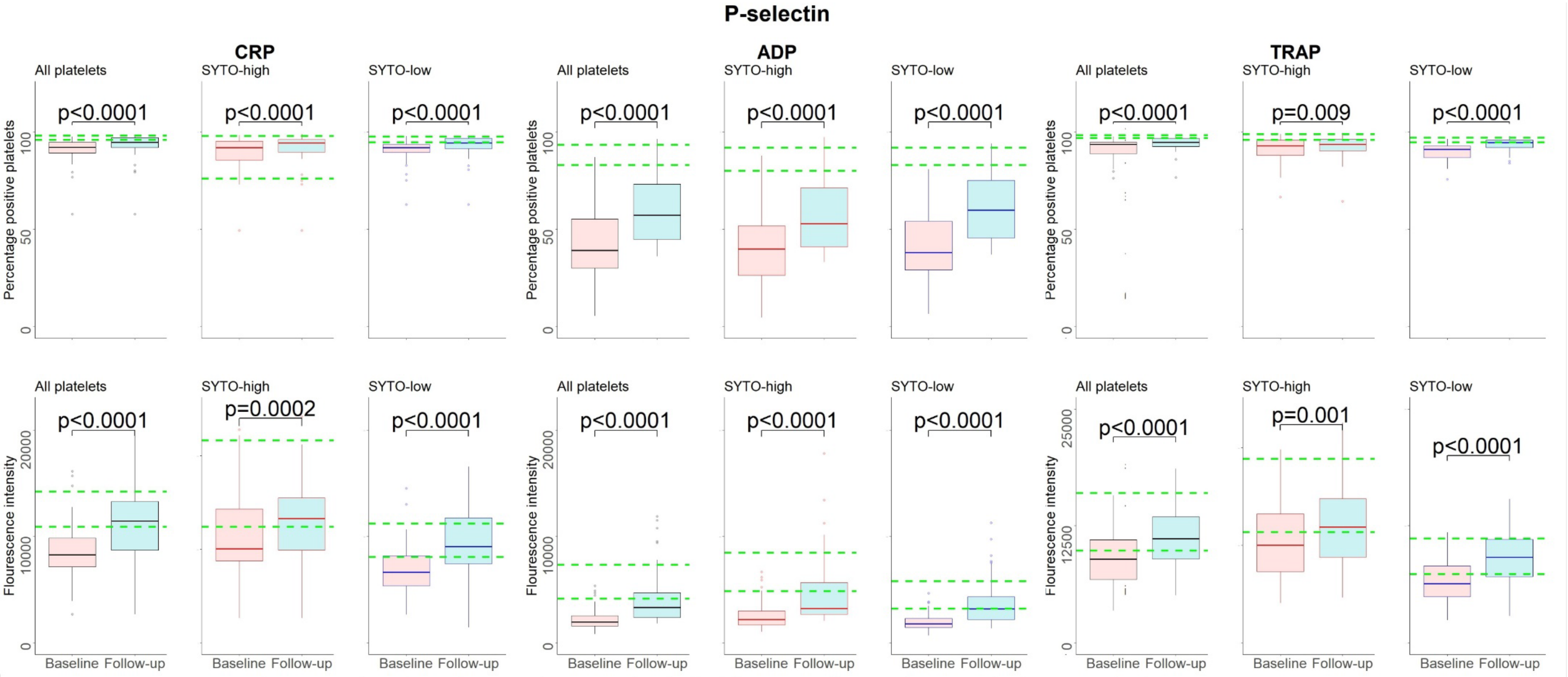

Box-plot represent the median and interquartile range. p-values for the difference were determined using paired Wilcoxon signed rank sum test. Y-axis shows either the percentage of reactivity marker positive platelets (top row) or the median fluorescence intensity of the reactivity markers on the platelet surface (bottom row). Green lines represent interquartile range in 50 health individuals. Adapted from Pedersen et al. [26]. Abbreviations: ADP: adenosine diphosphate, TRAP: thrombin-receptor-activating-peptide, CRP: collagen-related-peptide.

Supplementary Figure S4. Heatmap illustrating correlations between immature platelet markers and platelet reactivity at baseline in 44 patients with ST-segment elevation myocardial infarction (STEMI).

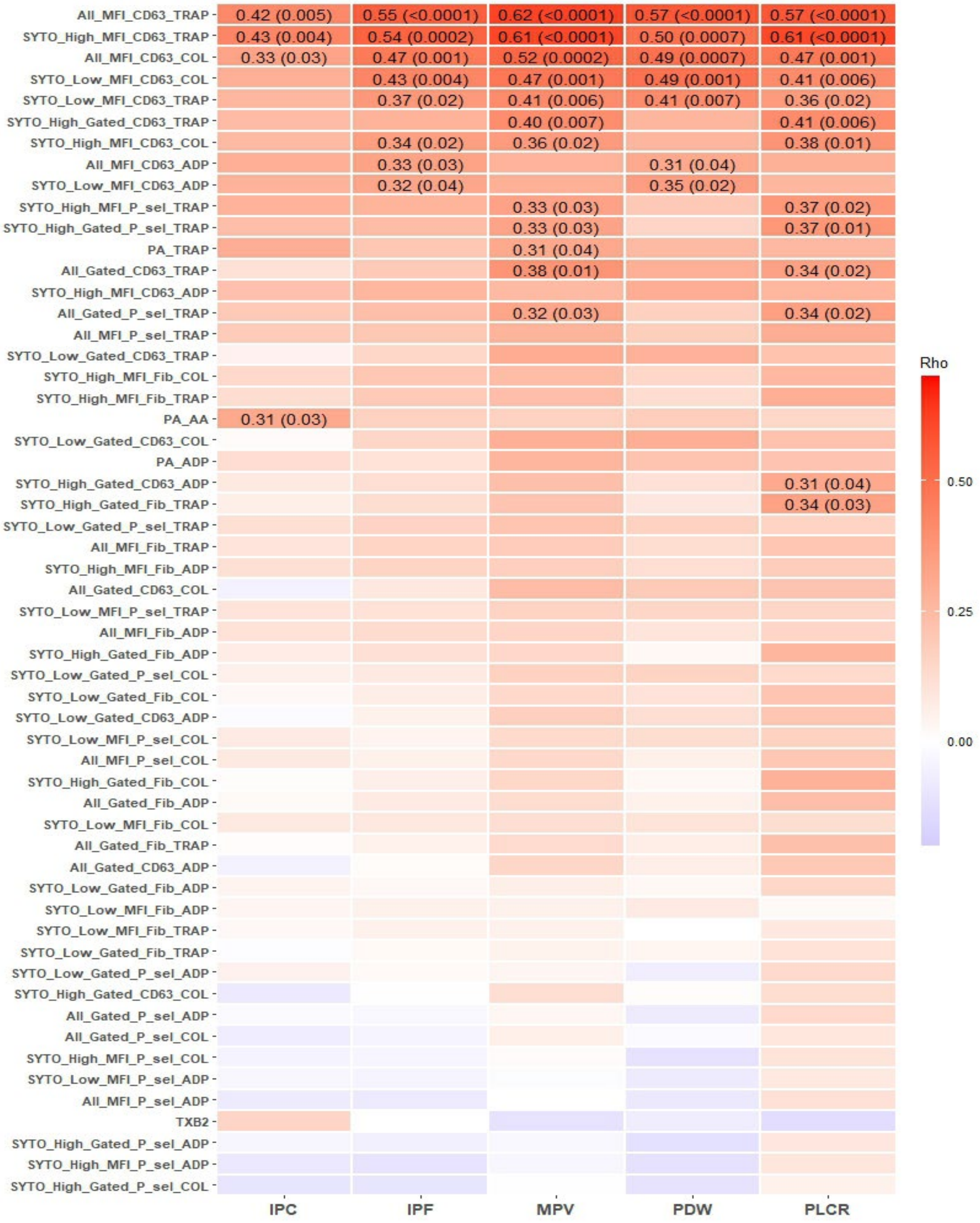

Statistics for significant correlations is indicated as Spearman's rho (p-value). Abbreviations: Rho: Spearman's Rho, All: all platelets, SYTO_High: 20% SYTO-13 high platelets, SYTO_Low: 20% SYTO-low platelets, IPC: immature platelet count, IPF: immature platelet fraction, MPV: mean platelet volume, PLCR: platelet large-cell-ratio, PDW: platelet distribution width, Gated: the percentage of platelets positive for the reactivity markers, MFI: median fluorescence intensity of the reactivity markers on the platelet surface, Anti-fib: anti-fibrinogen; P-sel: P-selectin; COL: collagen-related-peptide, TRAP: thrombin-receptor-acitivating-peptide-6, ADP: adenosine diphosphate, AA: arachidonic acid, TXB2: serum thromboxane B_2.

Supplementary Figure S5. Heatmap illustrating correlations between immature platelet markers and platelet reactivity at follow-up in 44 patients with ST-segment elevation myocardial infarction (STEMI).

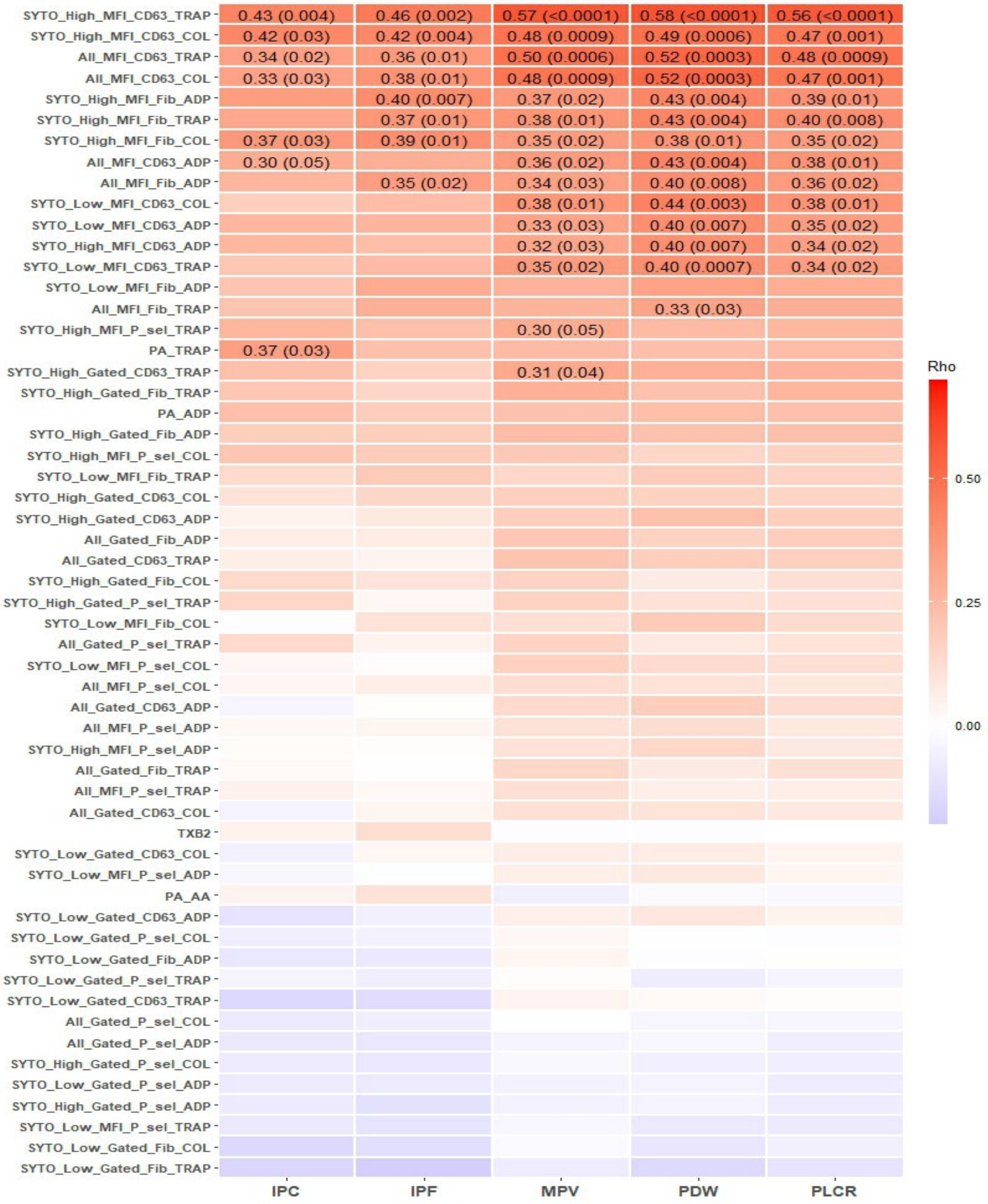

Statistics for significant correlations is indicated as Spearman's rho (p-value). Abbreviations: Rho: Spearman's Rho, All: all platelets, SYTO_High: 20% SYTO-13 high platelets, SYTO_Low: 20% SYTO-low platelets, IPC: immature platelet count, IPF: immature platelet fraction, MPV: mean platelet volume, PLCR: platelet large-cell-ratio, PDW: platelet distribution width, Gated: the percentage of platelets positive for the reactivity markers, MFI: median fluorescence intensity of the reactivity markers on the platelet surface, Anti-fib: anti-fibrinogen; P-sel: P-selectin; COL: collagen-related-peptide, TRAP: thrombin-receptor-acitivating-peptide-6, ADP: adenosine diphosphate, AA: arachidonic acid, TXB2: serum thromboxane B_2.

Supplementary Figure S6. Changes in serum thromboxane B2 levels from baseline to follow-up in 11 patients with ST-segment elevation myocardial infarction (STEMI) with thromboxane levels above 10 ng/mL at baseline.

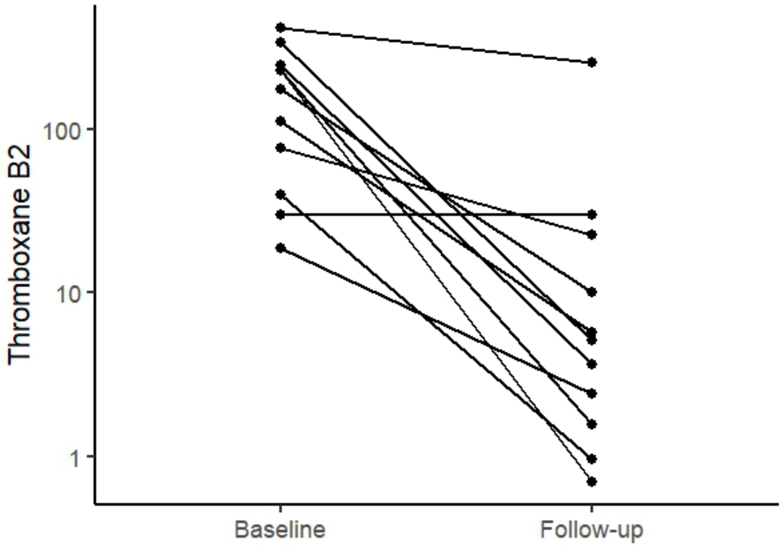

Supplementary Table S1. Difference in standard immature platelet markers and platelet function in patients with ST-segment elevation myocardial infarction (STEMI) at follow-up based on established cut-off (>46 aggregation units) using platelet aggregation with adenosine diphosphate (ADP) as agonist.

Group	Agonist	Marker	Measure	Patients below cut-off (n=32)	Patients above cut-off (n=9)
**Immature platelet count**				9.8 (6.5;15.7)	12.6 (7.7;18.2)
**Immature platelet fraction**				3.2 (2.4;5.0)	5.9 (2.5;7.0)
**Mean platelet volume**				9.4 (9.0;10.4)	10.0 (9.4;10.6)
**Platelet distribution width**				10.6 (9.5;12.10)	11.7 (10.7;12.70)
**Platelet-large-cell-ratio**				0.21 (0.17;0.29)	0.25 (0.21;0.30)
**Platelet reactivity using flow cytometry**	CRP	Anti-fib	%Gated<br>MFI	58 (49;65)<br>1,833 (1,712;2,147)	55 (50;62)<br>2,459 (1,915;2,733)
**Platelet reactivity using flow cytometry**	CRP	CD63	%Gated<br>MFI	71 (64, 76)<br>22,057 (18,665;26,246)	83 (79, 85)<br>28,244 (24,937;29,474)
**Platelet reactivity using flow cytometry**	CRP	P-sel	%Gated<br>MFI	95 (92;97)<br>11,652 (9,584;13,228)	97 (96;98)<br>13,300 (10,829;17,693)
**Platelet reactivity using flow cytometry**	TRAP	Anti-fib	%Gated<br>MFI	55 (39, 59)<br>1,671 (1,557, 1,942)	51 (39, 61)<br>2,273 (1,866, 2,737)
**Platelet reactivity using flow cytometry**	TRAP	CD63	%Gated<br>MFI	74 (69;79)<br>23,035 (20,544;27,805	81 (78;87)<br>28,370 (25,503;31,070)
**Platelet reactivity using flow cytometry**	TRAP	P-sel	%Gated<br>MFI	94.5 (93, 97)<br>12,284 (9,178;13,412)	95 (95;98)<br>13,438 (11,151;16,684)
**Platelet reactivity using flow cytometry**	ADP	Anti-fib	%Gated<br>MFI	29 (26;36)<br>1,586 (1,413;1,760)	40 (33;49)<br>2,224 (1,826;2,687)
**Platelet reactivity using flow cytometry**	ADP	CD63	%Gated<br>MFI	14 (12;22)<br>12,475 (10,368;15,401)	44 (39;58)<br>16,922 (15,321;20,468)
**Platelet reactivity using flow cytometry**	ADP	P-sel	%Gated<br>MFI	50 (45;64)<br>3,099 (2,476;3,729)	88 (75;90)<br>9,414 (4,385;10,766)
**Platelet aggrega-tion**	AA			131 (73;162)	305 (195;406)
**Platelet aggrega-tion**	TRAP			1,076 (885;1,227)	1,364 (1,228;1,400)
**Serum TXB2 levels**				2 (1;4)	10 (1;22)

All values are presented as median and interquartile range. Abbreviations: Anti-fib: Anti-fibrinogen, P-sel: P-selectin; %Gated: the percentage of platelets positive for the reactivity markers, MFI: median fluorescence intensity, CRP: collagen-related-peptide, ADP: adenosine diphosphate, TRAP: thrombin-receptor-activating-peptide, AA: arachidonic acid, TXB2; thromboxane B2.
